# Germination Triggers Substantial Changes in GABA, Polyphenol, Sugar, and Organic Acid Content of Commonly Consumed Legumes

**DOI:** 10.3390/plants15020242

**Published:** 2026-01-13

**Authors:** Daniela Pencheva, Desislava Teneva, Zornica Todorova, Manol Ognyanov, Ani Petrova, Vasil Georgiev, Mariya Pimpilova, Petko Denev

**Affiliations:** 1 Laboratory of Biologically Active Substances, Institute of Organic Chemistry with Centre of Phytochemistry, Bulgarian Academy of Sciences, 4000 Plovdiv, Bulgaria; daniela.klisurova@orgchm.bas.bg (D.P.); desislava.teneva@orgchm.bas.bg (D.T.); zornitsa.todorova@orgchm.bas.bg (Z.T.); manol.ognyanov@orgchm.bas.bg (M.O.); ani.petrova@orgchm.bas.bg (A.P.); mariya.pimpilova@orgchm.bas.bg (M.P.); 2 Centre of Competence “Sustainable Utilization of Bio-Resources and Waste of Medicinal and Aromatic Plants for Innovative Bioactive Products” (BIORESOURCES BG), 1000 Sofia, Bulgaria; 3 Laboratory Cell Biosystems, Department of Biotechnology, The Stephan Angeloff Institute of Microbiology, Bulgarian Academy of Sciences, 4000 Plovdiv, Bulgaria; vasgeorgiev@gmail.com

**Keywords:** legumes, germination, gamma-aminobutyric acid, polyphenols, nutritional profiles

## Abstract

This study investigated the effects of germination on gamma-aminobutyric acid (GABA), free sugars, organic acids, polyphenols, protein content, and antioxidant activity in six legumes (mung beans, Dobrudzha beans, white beans, brown lentils, red lentils and chickpeas). Seeds were germinated for 5 days at room temperature, with or without an initial freezing pretreatment at −18 °C for 20 h. Daily analysis revealed significant increases in GABA across all legumes, especially chickpeas, which showed an 18-fold rise to 210.5 mg/100 g dry weight (DW), alongside elevated glutamate decarboxylase activity. Total polyphenols increased 3.4-fold in white beans and chickpeas by day five. Antioxidant activity (ORAC) rose in parallel, reaching 123.8 and 83.3 µmol TE/g DW in germinated white beans and chickpeas, compared to 68.4 and 45.4 µmol TE/g DW in non-germinated controls. While protein content remained stable, levels of free sugars (notably maltose) increased during germination. Organic acids rose across all samples as well, with quinic acid being the most abundant and showing the sharpest increase. Initial freezing had a clear effect on enhancing GABA accumulation compared to non-treated seeds, but generally exerted neutral effects on other bioactive components. Overall, germination triggered biochemical transformations in seeds, enriching them with bioactive compounds and enhancing their nutritional and functional properties, with chickpeas emerging as a particularly rich source of GABA, polyphenols, and organic acids, supporting their potential in functional food development.

## 1. Introduction

Leguminous plants (Fabaceae) are an important source of plant-based proteins and bioactive compounds in human nutrition [1,2,3,4]. Their seeds contain high levels of proteins (10–45%), carbohydrates (30–80%), and fiber (3–40%) [5,6,7,8,9], along with essential minerals and phenolic compounds [10,11,12]. Despite their nutritional value, legume proteins are deficient in some essential amino acids and, with the exception of soybeans and peanuts, most species have low lipid content [2]. In addition to these nutrients, legumes also contain non-protein amino acids such as γ-aminobutyric acid (GABA), whose accumulation depends on physiological processes like germination and environmental stress.

Gamma-aminobutyric acid (GABA), a key inhibitory neurotransmitter in mammals, offers numerous health benefits, including calming effects, hypotensive action, sleep facilitation, stress and anxiety reduction, diabetes prevention, cholesterol regulation, and potential for cancer cell suppression, memory/learning improvement, and neurological condition treatment [13,14,15,16,17,18,19,20]. Found in various organisms, GABA is more concentrated in plant tissues than animal tissues, but with very few examples (i.e., tomatoes with GABA content up to 400 mg/100 g and potatoes up to 60 mg/100 g DW), the endogenous content of GABA in plant foods is relatively low [21]. As previously mentioned, legumes are a rich source of protein but are generally not particularly rich in GABA. For example, it has been reported that lentils contain approximately 5 mg of GABA per 100 g dry weight [22]. In comparison, mung beans exhibit a wider range, with GABA levels reported between 0.77 and 16.78 mg/100 g DW [23].

Germination conditions can influence the synthesis and thus the levels of bioactive compounds in sprouts, including GABA, polyphenols, flavonoids, sugars, and organic acids. To increase the GABA content of seeds, including legumes, methods like soaking, germination, and lactic acid fermentation can be employed [24,25]. These processes, including stress-inducing techniques (salt, drought, temperature), could boost GABA production, as it plays a protective role in plants [26,27,28]. Soaking and germination are simple yet effective methods that could improve nutrient profiles (protein, vitamins, sugars, GABA), flavor, and structure while reducing anti-nutritional factors like phytic acid [2,29]. It has been revealed that germination could increase slightly protein content in mung beans (27.5 to 30 g/100 g DW) and chickpeas (23.64 to 25.53 g/100 g DW), and fibre in chickpeas (3.82 to 5.21 g/100 g DW), while decreasing fat [11,30]. It also elevated free sugars (especially in soybeans), tocopherols, and vitamin C (e.g., mung beans: 11.69 to 285 mg/100 g DW) [31]. Total polyphenol content (e.g., chickpeas: 12 to 19 mg/100 g; mung beans: up to 966.4 mg/100 g) [31,32] and individual flavonoids and isoflavones could also be affected by germination [33]. According to Luo et al. (2018), GABA levels in soybean increased after germination, rising from 5 mg/100 g DW on day 0 to 10 mg/100 g DW on day 1, and further to 26 mg/100 g DW on day 5 [34]. Other authors published that GABA was not present in the raw seeds of beans, lentils, and peas, but its content rose after germination to 44, 32, and 104 mg/100 g DW, respectively [35].

Cold stress could trigger the accumulation of specific molecules that help protect plants, including polyphenols, sugars, and amino acids [36,37,38]. GABA also has a protective role in plants, and its quantity increases during stress conditions. This amino acid plays a key role in osmoprotection, enhanced photosynthetic capacity, and reduces stress by activating the antioxidant defense system enzymes like superoxide dismutase, catalase, ascorbate peroxidase, glutathione peroxidase, and glutathione S-transferase [38,39]. It has been shown that cold stress could increase GABA levels in different chickpea genotypes by 46–58% in cold-tolerant genotypes and by 10–18% in cold-sensitive ones [40]. Another study revealed that germination of soya under cold stress (−18 °C) increases GABA content more than seven times, from 0.29 to 2.15 mg/g DW [41].

Physiological and biochemical transformations during germination are strongly influenced by pre-germination stressors, particularly those introduced through various physical treatments. Considering the vast diversity of legume species cultivated and consumed worldwide, understanding how such treatments affect their nutritional and phytochemical profiles is of significant interest. In this context, the present study aimed to investigate the effect of germination, with and without prior freezing, on key nutritional and bioactive components of commonly consumed legumes in Bulgaria, including chickpeas, mung beans, white beans, Dobrudzha beans, brown lentils, and red lentils. The parameters assessed included GABA content, protein levels, sugar content and composition, polyphenol content and composition, organic acid profiles, and antioxidant activity. The findings identify specific germinated legumes with enhanced levels of bioactive compounds, highlighting their potential as functional food ingredients.

## 2. Results and Discussion

### 2.1. Effect of Germination on GABA and Protein Content of Legumes

**Figure 1:**
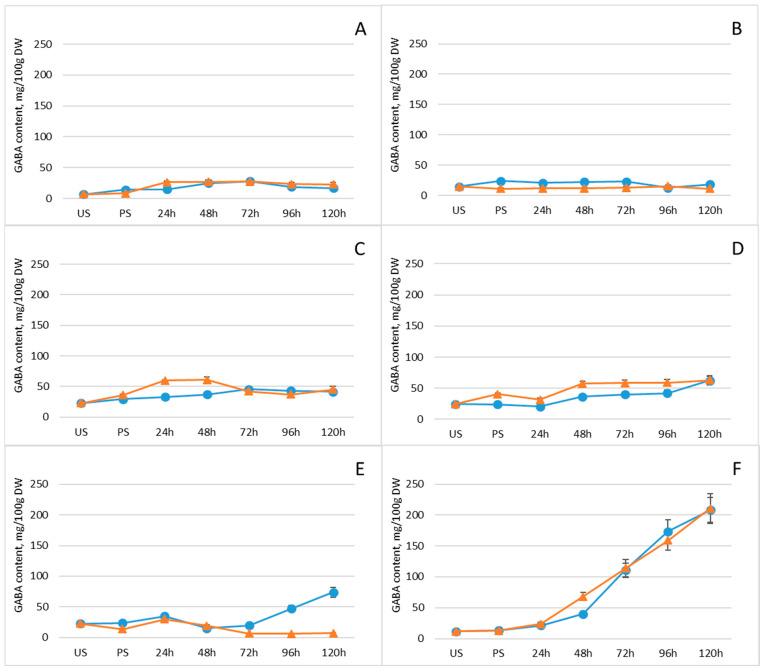
Changes in GABA content (mg/100 g DW) of legumes after soaking and germination with (orange line) and without (blue line) freezing pretreatment: (**A**) mung beans, (**B**) Dobrudzha beans, (**C**) white beans, (**D**) brown lentils, (**E**) red lentils, (**F**) chickpeas. Values represent mean ± standard deviation of two biological replicates. Statistical differences between time points or treatments were evaluated using one-way ANOVA, followed by Student’s *t*-test for pairwise comparisons.

Germination conditions can influence the synthesis and thus the levels of bioactive compounds in sprouts, including GABA, polyphenols, flavonoids, sugars, and organic acids [42]. Comparative analysis of GABA levels in mung beans, white beans, Dobrudzha beans, brown lentils, red lentils, and chickpeas revealed that germination increased GABA content in all tested legumes, although the extent and peak levels of accumulation varied among species (Figure 1, Supplementary Table S1). GABA increase was the most significant (18-fold, *p* < 0.05) in chickpeas reaching levels of 210.5 mg/100 g DW with freeze pretreatment and 208.2 mg/100 g DW, respectively. Overall, GABA accumulation was faster in the first days in freeze-pretreated white beans, brown lentils and chickpeas, indicating that this particular pretreating before germination could be more effective in boosting GABA levels compared to conventional germination. However, these difference disappeared with the time and the only significant difference between groups was observed in red lentils on day five, where GABA content (74 ± 8.1 mg/100 g) was 10-fold higher in the non-pretereated group.

Although germination rates differ among legume species—typically requiring approximately 3–4 days in lentils and 5–6 days in chickpeas, a standardized 5-day germination period was selected to enable meaningful comparison across species at a comparable developmental stage. This duration ensured that all legumes had visibly initiated germination, thereby providing a consistent basis for sampling. While inherent differences in seed morphology and physiology may influence specific biochemical responses, the adoption of a fixed germination interval permitted an objective assessment of metabolic changes under controlled and comparable conditions.

**Figure 2:**
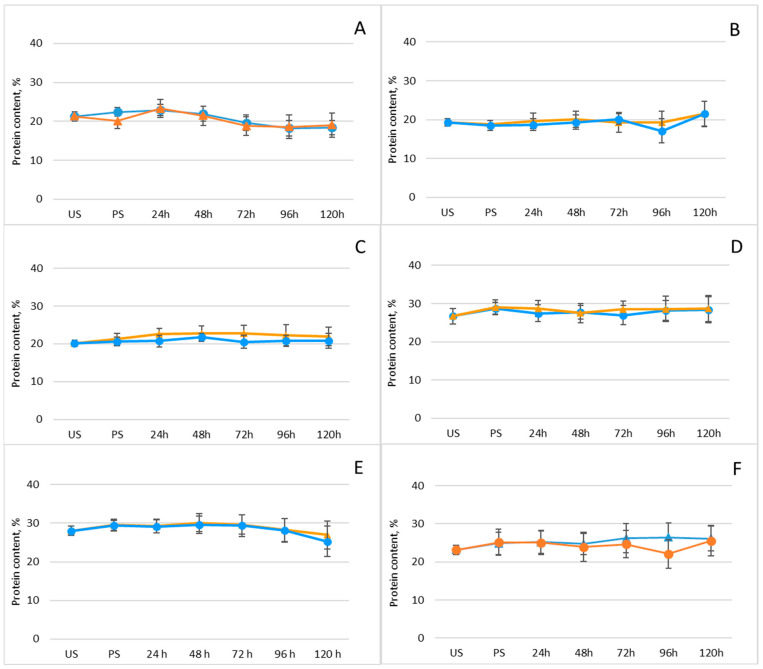
Changes in protein content (%) of legumes after soaking and germination with (orange line) and without (blue line) freezing pretreatment: (**A**) mung beans, (**B**) Dobrudzha beans, (**C**) white beans, (**D**) brown lentils, (**E**) red lentils, (**F**) chickpeas. Results are presented as mean values ± standard deviations. Values represent mean ± standard deviation. Statistical differences between time points or treatments were evaluated using one-way ANOVA, followed by Student’s *t*-test for pairwise comparisons.

Legumes are well known for their high protein content and for supplying essential amino acids that serve as fundamental building blocks of the human body. This substantial protein content also renders legumes a promising substrate for GABA production [43]. For this reason, the effect of germination on the total protein content of the investigated legumes was examined (Figure 2; Supplementary Table S1).

As shown, protein levels remained largely unchanged, regardless of whether the seeds underwent an initial freezing pretreatment prior to germination. Although no significant variation in total protein content was observed, the observed increases in GABA content in our study are entirely consistent with previous reports of GABA accumulation during legume germination. For instance, germination has been shown to markedly elevate GABA concentrations in chickpeas—from 6.42 to 245.76 mg/100 g [44]. In another study, mung beans soaked for 4 h at 40 °C and pH 5.5, followed by 7 h of germination, exhibited a 10-fold increase in GABA content, reaching 1677 mg/kg [45]. Similarly, soaking mung beans in tap water (1:5 *w*/*v*) for 12 h at room temperature and germinating them for 36 h resulted in a 15-fold increase in GABA content (3.93 mg/g) relative to ungerminated controls [46]. Substantial GABA accumulation has also been reported in adzuki beans, with an 18.52-fold increase [47], as well as in black soybeans, showing a 3.73-fold enhancement [48]. Furthermore, several studies suggest that freezing or freeze–thaw treatments can stimulate GABA biosynthesis by disrupting cellular membranes and enhancing solute redistribution. Yang et al. demonstrated a significant increase in GABA content in soybean sprouts following thawing, indicating that GABA formation predominantly occurs during the thawing phase [49].

**Figure 3:**
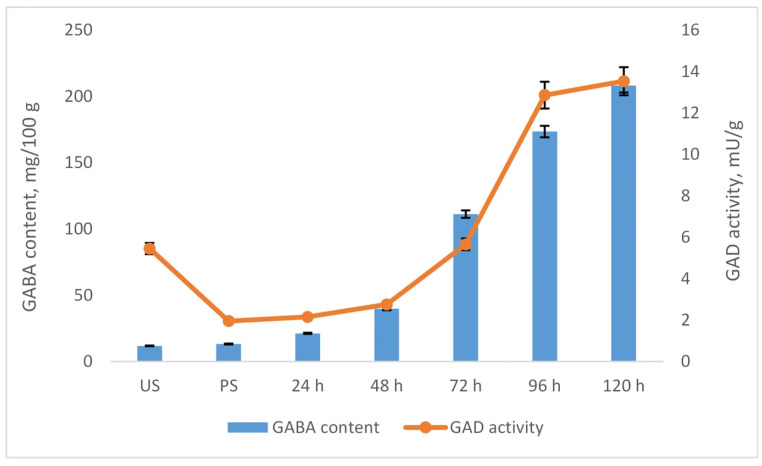
Correlation between GABA content and GAD activity in chickpeas during germination. Results are presented as mean values ± standard deviations. Values represent mean ± standard deviation.

To elucidate the mechanism underlying the GABA accumulation observed during germination, we analyzed the activity of glutamate decarboxylase (GAD) in chickpea samples, as this enzyme is directly responsible for GABA biosynthesis. Figure 3 presents the temporal profiles of GABA content and GAD activity in samples germinated without prior freezing pretreatment. As shown, a strong correlation (r = 0.93) was observed between GAD activity and GABA accumulation throughout the germination period, underscoring the central role of GAD in GABA synthesis. The parallel increase in GAD activity and GABA content suggests that GABA accumulation during germination is primarily driven by enhanced GAD-mediated glutamate decarboxylation.

### 2.2. Effect of Germination on Phenolic Content and Composition, and Antioxidant Activity of Legumes

**Figure 4:**
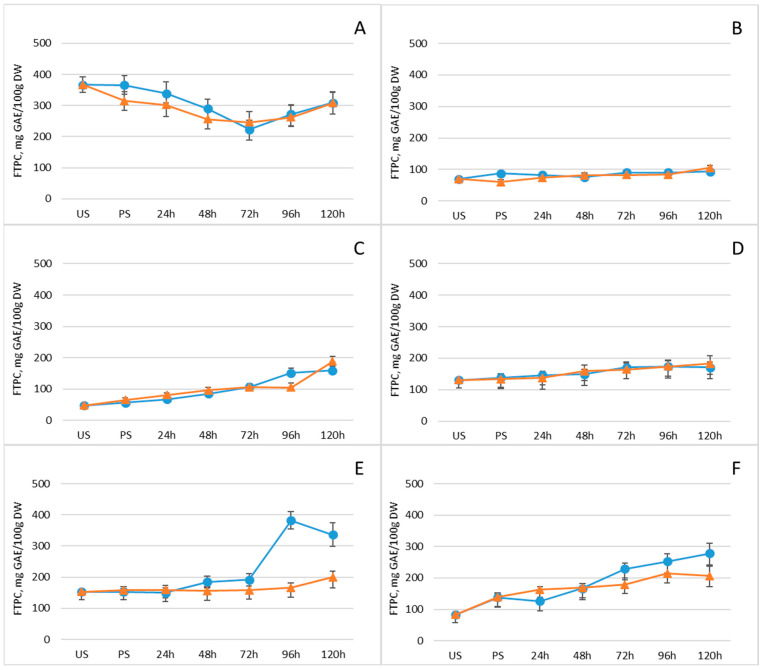
Changes in free total polyphenol content (mg GAE/100 g DW) of legumes after soaking and germination with (orange line) and without (blue line) freezing pretreatment: (**A**) mung beans, (**B**) Dobrudzha beans, (**C**) white beans, (**D**) brown lentils, (**E**) red lentils, (**F**) chickpeas. Values represent mean ± standard deviation. Statistical differences between time points or treatments were evaluated using one-way ANOVA, followed by Student’s *t*-test for pairwise comparisons.

Polyphenols are crucial antioxidants that play a central role in neutralizing free radicals, regulating plant metabolism, and protecting tissues from ultraviolet radiation. Consequently, they help prevent oxidation in food matrices and mitigate oxidative damage in the human body [50]. Their levels in legumes, like those of other bioactive compounds, can be modulated through germination. Figure 4 and Supplementary Table S1 present the free total polyphenol content (FTPC) across the legume samples, while Table 1 provides quantitative data on the major individual phenolic compounds in control seeds, post-soaking, and on the fifth day of germination. In five of the six investigated legumes, total polyphenol content increased progressively after soaking, reaching maximum levels on day five of germination. In contrast, mung beans showed an initial decline in polyphenol levels from soaking through day three, followed by a modest increase by day five. Differences between freezing-pretreated and non-pretreated samples were generally minor; however, seeds subjected to freezing at −18 °C prior to germination tended to exhibit slightly lower polyphenol levels, suggesting a mild inhibitory effect on phenolic accumulation. The most pronounced increase in total polyphenol content (~230%) occurred in white beans and chickpeas, with chickpeas showing the highest absolute value by day five, reaching 278 mg/100 g DW. Among the individual phenolic compounds, catechin levels rose substantially in all legumes by the fifth day of germination. Chickpeas showed particularly notable increases in quercetin, from 80.0 ± 8 µg/g DW to 1540 ± 150 µg/g DW (*p* < 0.05), and in protocatechuic acid, from 23 ± 2 µg/g DW to 64 ± 6 µg/g DW, emphasizing their potential as a rich source of antioxidant phenolics following germination.

**Figure 5:**
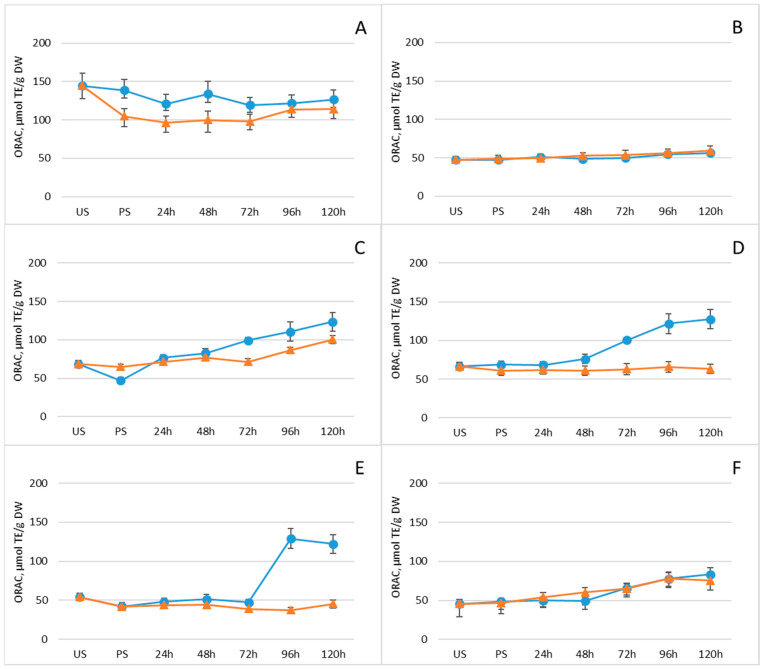
Changes in ORAC antioxidant activity (μmol TE/g DW) of legumes after soaking and germination with (orange line) and without (blue line) freezing pretreatment: (**A**) mung beans, (**B**) Dobrudzha beans, (**C**) white beans, (**D**) brown lentils, (**E**) red lentils, (**F**) chickpeas. Values represent mean ± standard deviation. Statistical differences between time points or treatments were evaluated using one-way ANOVA, followed by Student’s *t*-test for pairwise comparisons.

Given the substantial contribution of phenolic compounds to the antioxidant properties of plants and food products, ORAC values were determined at each time point during germination. Figure 5 and Supplementary Table S1 display the ORAC antioxidant activity of the legume samples. Overall, germination enhanced ORAC activity in all legumes, with a continuous increase observed throughout the germination period. This trend parallels the pattern observed for total polyphenol content in both experimental groups (Figure 4 and Table 1). In the group without freezing pretreatment, ORAC activity continued to rise steadily across the entire germination timeline.

Previous research has shown that germination can influence antioxidant activity in legumes such as soybeans, lentils, and vicia, although the relationship with antioxidant compound content is not always direct or consistent [51]. Numerous studies have reported increases in total polyphenol content during germination, including in mung beans, black beans, and soybeans [52,53]; lentils [54]; faba beans, chickpeas, lentils, and fenugreek seeds [55]; and lupine seeds [56]. Conversely, some authors have documented declines in phenolic content during germination, as observed in kidney beans and lentils [57,58]. Aguilera et al., for example, reported a significant reduction in total polyphenols in germinating lentil seeds, whereas kidney beans showed no notable change [59]. Salem et al. demonstrated variability in bioactive compound levels and antioxidant activity among several legume species [55]. Faba beans, chickpeas, lentils, and fenugreek seeds exhibited 33.65, 57.94, 60.39, and 56.14 mg gallic acid/g of total phenols; 6.39, 5.54, 5.54, and 6.58 mg quercetin/g of total flavonoids; and antioxidant activities of 95.16%, 72.80%, 81.65%, and 90.69%, respectively. In contrast, broad beans (5.04 mg gallic acid/g) and lupine seeds (5.38 mg gallic acid/g) contained lower total polyphenol levels than faba beans (7.11 mg gallic acid/g) and lupine seeds (8.56 mg gallic acid/g) [56,60]. These discrepancies may arise from differences in species, cultivation and storage conditions, or the extraction and analytical methods employed.

It should be noted that the presented results for total polyphenol content, individual phenolics, and resulting antioxidant activity refer only to the free phenolic fraction, as the extraction procedure used does not release bound polyphenols. This distinction is important because previous studies have shown that sprouting predominantly increases the free phenolic fraction and the associated antioxidant activity, rather than the bound fraction [61].

### 2.3. Effect of Germination on Free Sugar Content and Composition of Legumes

Carbohydrates, including free sugars, play a fundamental role in shaping both the nutritional value and sensory attributes of legumes. Consequently, elucidating the impact of germination and freezing on free sugar levels is essential for understanding the metabolic dynamics of these seeds. Table 2 summarizes the changes in free sugar composition (fructose, sucrose, galactose, and maltose) across all studied legumes. Among these, maltose exhibited the most pronounced accumulation during germination, becoming the dominant sugar and the only detectable monosaccharide in the bean varieties. Its concentration increased between two- and ten-fold across all legumes, representing the most substantial shift among all quantified parameters. A general pattern emerged in which free sugar levels rose more markedly in samples without freezing pretreatment. In mung beans, freezing initially suppressed fructose, galactose, and maltose levels; however, these sugars increased as germination progressed. Another notable observation was the rise in fructose content in legumes where it was present, particularly mung beans, brown lentils, red lentils, and chickpeas. Chickpeas were unique in that sucrose was detectable only in this species, reaching a maximum of 560 ± 46 mg/100 g DW on the second day of germination following freezing pretreatment. This sucrose accumulation may reflect cold-induced suppression of cell wall-bound invertase activity, an enzyme responsible for hydrolyzing sucrose into glucose and fructose. Reduced invertase activity under cold conditions may lead to sucrose retention, potentially linked to impaired tapetal function, which in turn restricts carbohydrate transport to developing microspores.

Germination, irrespective of prior freezing treatment, resulted in an overall increase in free sugar content, consistent with previous reports in legumes [62,63,64]. Martín-Cabrejas et al. found that germination elevated total soluble sugars in all legume samples while substantially reducing raffinose-family oligosaccharides [65]. During germination, the activation and release of hydrolytic enzymes, such as α-amylase, β-amylase, and α-glucosidase, drive the degradation of starch and other polysaccharides into simpler sugars, including maltose, fructose, sucrose, dextrins, and glucose [66,67,68,69]. This enzymatic activity likely accounts for the pronounced increase in maltose observed across all studied legumes. Interestingly, glucose was not detected in any of the samples, despite the expectation that starch degradation during germination would liberate significant quantities of glucose [70].

### 2.4. Effect of Germination on Organic Acid Content and Composition of Legumes

Organic acids are important contributors to the palatability and health-promoting properties of foods. Substantial changes in organic acid content were observed among the studied samples, with all detected acids increasing throughout the entire germination period (Table 3). Research on organic acid dynamics during legume germination remains limited, particularly under freezing pretreatment. To our knowledge, this study is the first to quantify their accumulation following such a treatment. In general, organic acid content decreased during soaking and then rose rapidly and consistently as germination progressed. As shown in Table 3, quinic acid was the predominant organic acid across all legumes, which also contained oxalic, tartaric, shikimic, fumaric, and malic acids in smaller quantities. Mung beans subjected to initial freezing exhibited the highest quinic acid levels (8730 ± 710 mg/100 g DW). In lentils, peak quinic acid content varied by type and growing conditions: brown lentils reached their maximum on the third day of germination without freezing (4650 ± 460 mg/100 g DW), while red lentils peaked on the fourth day, with higher values observed after freezing (3830 ± 310 mg/100 g DW). Chickpeas reached their maximum quinic acid levels on the fifth day of germination in both groups—without (6890 ± 580 mg/100 g DW) and with (6370 ± 600 mg/100 g DW) freezing pretreatment.

Levels of several organic acids, such as malic, citric, lactic, and fumaric acids, are known to increase during germination [71,72,73]. Early germination stages in mung beans are characterized by marked metabolic shifts, particularly in organic acid metabolism, with malic acid exhibiting notable changes [74]. More recently, Chen et al., using NMR spectroscopy, demonstrated that six days of germination increased numerous metabolites in mung beans, including raffinose, maltose, glucose, α-ketoglutaric acid, acetic acid, GABA, galactose, arabinose, and sucrose [75]. Perchuki et al. reported that mung bean sprout leaves, grown either in darkness or light, contain a rich and diverse metabolite profile beneficial to human health, identifying more than 100 compounds, including several organic acids such as oxalic, lactic, pyruvic, citric, succinic, fumaric, and malic acids [73].

Understanding the accumulation of organic acids during germination requires consideration of their physiological roles in the developing seed. These acids perform essential functions in the embryo and radicle, contributing to osmotic regulation, energy metabolism, and root exudation into the rhizosphere. Malic and citric acids facilitate the solubilization of phosphorus and micronutrients required for early root and shoot growth, and they participate in aluminum detoxification in acidic soils, supporting legume tolerance under adverse environmental conditions [76,77]. Oxalic acid, exuded particularly by legume roots such as soybean and cowpea, modulates rhizosphere pH and promotes the proliferation of beneficial microorganisms, including rhizobia and phosphate-solubilizing bacteria [78]. Within seed tissues, fumaric and malic acids act as intermediates of the citric acid cycle, sustaining metabolic activity and enhancing resilience against oxidative stress during germination [79]. Quinic and shikimic acids, though less studied in legumes, likely serve as intermediates in phenolic biosynthesis, contributing to seedling defense and establishment [80]. Tartaric acid may assist in metal chelation and root signaling, although its roles in legumes require further clarification [81]. From a nutritional perspective, these acids collectively exhibit antioxidant, antimicrobial, and mineral-chelating properties. Malic and fumaric acids support mitochondrial energy production and reduce fatigue; oxalic and tartaric acids contribute antimicrobial and anti-inflammatory effects; quinic and shikimic acids serve as precursors for antiviral and antioxidant phenolics; and the combined presence of these acids supports gut health, nutrient absorption, and metabolic balance [82].

Notably, ascorbic acid (vitamin C) was not detected in measurable amounts in any of the legumes, either before or during germination. Because extraction was performed at neutral pH, endogenous ascorbate oxidase may have oxidized reduced ascorbic acid to dehydroascorbic acid, potentially leading to a slight underestimation of its true levels in germinated legumes.

It should also be noted that to monitor changes in the chemical composition of legumes, only one genotype per species was analyzed in this study. Therefore, the conclusions apply specifically to the cultivars examined. Differences observed among samples of the same species indicate that the metabolic effects of germination conditions are genotype-dependent. Accordingly, species-level generalizations cannot be drawn from a single genotype, and future research should incorporate multiple genotypes to enable broader conclusions across legume species.

## 3. Materials and Methods

### 3.1. Chemicals

All analytical materials, reagents, and standards of flavonoids and phenolic acids (gallic acid, protocatehuic acid, (+)-catechin, chlorogenic acid, vanillic acid, caffeic acid, syringic acid, (−)-epicatechin, p-coumaric acid, ferulic acid, salicylic acid, rutin, hesperidin, rosmarinic acid, quercetin, kaempherol), sugars (sucrose, fructose, galactose, maltose) and organic acids (quinic, shikimic, fumaric, malic, oxalic and tartaric), were purchased from Sigma-Aldrich (Darmstadt, Germany).

### 3.2. Plant Materials

Following legumes were used in the study: mung beans (*Vigna radiate* L.), produced in Argentina, Batch LC150124/01; Dobrudzha beans (*Phaseolus vulgaris* L., var. Dobrudzhanski fasul), produced in Bulgaria, purchased from a marketplace; white beans (*Phaseolus vulgaris* L., var. Bial fasul), produced in Bulgaria, Batch LC090124/01; Brown lentils (*Lens culinaris* Medik.) produced in Bulgaria, Batch LC030124/01; Red lentils (*Lens culinaris* Medik.), produced in Turkey, Batch 29306/02052025.

### 3.3. Germination Conditions

Seeds of each legume were divided into two experimental groups. The first group underwent a standard imbibition and germination procedure for 5 days (120 h) without freezing pretreatment, to evaluate natural biochemical changes. The second group was pretreated by freezing at −18 °C for 20 h before soaking and germination for 5 days (120 h). Each group comprised six sample clusters, representing treatment (soaking) and time points, denoted as: PS (post-soaking), 24 h, 48 h, 72 h, 96 h, and 120 h. In addition, a single untreated seed cluster—US (untreated seeds) served as a common control for both groups. Each sample cluster contained 25 g of seeds, corresponding approximately to 480 seeds of mung bean, 50 seeds of Dobrudzha bean, 54 seeds of white bean, 353 seeds of brown lentil, 782 seeds of red lentil, and 64 seeds of chickpea. Controlled germination was performed by first steeping each 25 g cluster in 0.1% (*v*/*v*) sodium hypochlorite solution for 10 min, followed by three rinses with distilled water. Seeds were then soaked in 200 mL distilled water for 12 h at room temperature in darkness to initiate germination. After soaking, the water was drained, and the PS clusters were separated for freeze-drying and analysis. Seeds from the remaining five clusters for each legume were evenly distributed on sieves and incubated in darkness to simulate natural conditions (under soil cover), with moisture maintained by watering twice daily. One 25 g cluster from each group and legume was collected every 24 h for further analysis. To arrest metabolic activity, all samples collected on a given day were frozen immediately at −18 °C in a sealed bag and subsequently freeze-dried using a lyophilizer (Alpha 1–4 LDplus, Martin Christ Freeze Drying Systems GmbH, Osterode am Harz, Germany). Following lyophilization, the samples were ground into a fine powder.

### 3.4. High-Performance Liquid Chromatography Analysis of GABA

The determination of GABA was carried out as described in our previous study [21]. In brief, 1 g of the powdered sample was extracted with 75% ethanol (15 mL) on a magnetic stirrer for 1 h at room temperature. The filtered extracts were mixed with 0.1 M sodium hydrogencarbonate buffer (pH 8.7) and further derivatized (55 °C, 1 h) with a freshly prepared acetone solution of dansyl chloride. The quantification of GABA was performed using the UHPLC system Nexera *i* LC 2040 C Plus (Shimadzu Corporation, Kyoto, Japan). The system was equipped with a binary pump, an Accucore (Thermo Fisher Scientific) C18 (21 mm × 150 mm, 26 µm) column, and a UV-VIS detector. All separation conditions and the composition of the mobile phase were the same as described by Pencheva et al. [21].

### 3.5. Protein Content

The crude protein content of the initial plant samples was evaluated by the micro-Kjeldahl method. The determination of nitrogen expressed as ammonia content of the digested sample was performed by the acetylacetone–formaldehyde colorimetric method using ammonium sulfate as a standard [83]. The nitrogen content was converted into crude protein by multiplying by a factor of 6.25. The protein content of the samples during germination was estimated by employing the alkaline copper (biuret) reagent [84]. In brief, the ground samples were initially extracted with acetone (1 g with 80 mL) to overcome the interfering effects of lipids. Furthermore, about 1.00 g of dried seed material was extracted with 0.4% (*w*/*v*) Na_2_CO_3_ solution in a 50 mL volumetric flask. After shaking for 1 h at 37 °C, the mixture was centrifuged at 5000× *g* for 10 min. Two mL of supernatant were transferred to a test tube, and 8 mL of biuret reagent were added. Then the mixture was allowed 30 min for full color development before measuring absorbance at 550 nm. The method was standardized using the same sample of known protein content as determined by the Kjeldahl procedure. To construct the regression lines between biuret absorbance values and Kjeldahl protein values for each sample, a sample containing 250 mg protein was weighed out and extracted as described above. A convenient volume of supernatant, containing between 2.5 and 10.0 mg protein, was finally diluted to 2 mL and run as a sample. Standard curves for all legumes are shown in Supplementary Figure S1.

### 3.6. Glutamate Decarboxylase (GAD) Activity Assay

The GAD enzyme was extracted according to the method described by Yu et al. [85], using a 50 mM phosphate buffer (pH = 5.7) containing 2 mM EDTA, 0.15 M NaCl, 0.2 mM pyridoxal phosphate, and 0.2% (*v*/*v*) β-mercaptoethanol (buffer A). The extraction was performed at a 1:5 (*w*/*v*) ratio and the mixture was homogenized using a vortex mixer for 15 min at room temperature. After centrifugation at 14,000× *g* for 20 min at 4 °C, 0.3 mL of the supernatant was mixed with 0.2 mL of 50 mM phosphate buffer (pH = 5.7) containing 0.2 mM pyridoxal phosphate and 100 mM L-glutamic acid (buffer B). The samples were incubated at 40 °C for 120 min and GABA produced was determined following the HPLC method described by Pencheva et al. [21]. Together with the test samples, control samples were run, replacing buffer B with water (0.2 mL) in order to evaluate the quantity of GABA in the enzyme (buffer A) extracts. Enzyme activity was expressed as mU/g. One standard unit of GAD activity is defined as that amount of enzyme that catalyzes the production of 1 μmol product (GABA) per minute under the specified assay conditions.

### 3.7. Total Polyphenolic Content

Free phenolic fraction from freeze-dried and ground samples (1 g) was extracted with 15 mL of solvent containing 75% ethanol at room temperature on a magnetic stirrer for 1 h. Then, the sample was centrifuged (6000× *g*, 20 min), and the supernatant was collected for amalysis of free total polyphenols content and antioxidant activity. Polyphenol content was determined according to the method of Singleton and Rossi with the Folin–Ciocalteu reagent [86]. Gallic acid (10–200 µg/mL) was used as a standard.

### 3.8. High-Performance Liquid Chromatography of Phenolic Components

Quantification of phenolic compounds in extracts (obtained in Section 3.7) was performed by HPLC (Waters 1525) system with UV-VIS detector (Waters 2487) as described previously [87]. In brief, 20 µL of the extract were injected into C18 column (Supelco Discovery HS; 5 μm, 25 cm × 4.6 mm) (Merck KGaA, Darmstadt, Germany) and gradient elution (1.0 mL/min flow rate) by using a 1% acetic acid (Solvent A) and methanol (Solvent B) was applied. The absorption at λ = 280 nm was used to detect gallic, protocatechuic, vanillic, syringic, *p*-coumaric, and salicylic acids, (+)-catechin, (+)-epicatechin, and hesperidin. Rosmarinic, chlorogenic, caffeic, and ferulic acids, rutin, quercetin, and kaempferol were detected at λ = 360 nm. Quantification was performed using the retention times and calibration curves of external standards.

### 3.9. In Vitro ORAC Antioxidant Activity

Oxygen Radical Absorbance Capacity was measured according to [88] with some modifications [89]. Solutions of AAPH, FL, and trolox were prepared in a phosphate buffer (75 mmol/L, pH 7.4). Samples were diluted in the phosphate buffer as well. Reaction mixture (total volume 200 μL) contained FL—(170 μL, final concentration 5.36 × 10^−8^ mol/L), AAPH—(20 μL, final concentration 51.51 mmol/L), and sample—10 μL. The FL solution and sample were incubated at 37 °C for 20 min directly in a microplate reader, and AAPH (dissolved in buffer at 37 °C) was added. The mixture was incubated for 30 s before the initial fluorescence was measured. After that, the fluorescence readings were taken at the end of every cycle (1 min) after shaking. For the blank, 10 μL of phosphate buffer was used instead of the extract. Trolox solutions (3.125; 6.25; 12.5; 25 and 50 μmol/L) were used for defining the standard curve. ORAC was measured using a FLUOstar OPTIMA plate reader (BMG Labtech, Germany), excitation wavelength of 485 nm and an emission wavelength of 520 nm. ORAC values were expressed in µmol TE per gram DW.

### 3.10. High-Performance Liquid Chromatography Analysis of Free Sugars

Extraction and analysis of free sugars were performed according to the methodology of Ognyanov et al., 2024 [90]. The chromatographic separation and quantification of free sugars were conducted on a Nexera-*i* LC2040C Plus UHPLC system (Shimadzu Corporation, Kyoto, Japan), coupled with a Zorbax Carbohydrate column (4.6 mm × 150 mm, 5 μm) and Zorbax Reliance Cartridge guard-column operating at 35 °C. Approximately 1 g of the freeze-dried samples was extracted with 30 mL of distilled water for 1 h at 30 °C on a shaking thermostatic water bath. After centrifugation at 6000× *g*, the supernatants were used for UHPLC analysis of sugars. The samples were injected (10 μL) and eluted with a mobile phase composed of a mixture of acetonitrile/H_2_O (80/20 *v*/*v*) at a flow rate of 1.0 mL/min. The eluate was monitored using a refractive index detector RID-20A (cell temperature 40 °C). The concentration of sugars in the sample was deduced using a calibration curve built by plotting the peak area against different concentrations for each sugar. The different sugars in the sample were confirmed by a comparison of retention time with that of the standards [90].

### 3.11. High-Performance Liquid Chromatography Analysis of Organic Acids

Extraction and analysis of organic acids were performed according to Ognyanov et al. [90]. UHPLC determination of organic acids was performed on a Nexera-*i* LC2040C Plus system (Shimadzu Corporation, Kyoto, Japan) equipped with a UV detector (210 nm). The separation was conducted on a Shim-pack GIST C18 (5 µm, 250 mm × 4.6 mm) column at 25 °C and a flow rate of 1.0 mL/min. Freeze-dried samples (approximately 1 g) were extracted with 30 mL of distilled water for 1 h at 30 °C in a shaking water bath. Following centrifugation at 6000× *g*, the supernatants were analyzed for organic acids using UHPLC. The sample was eluted isocratically using a 25 mM solution of K_2_HPO_4_ in water as a mobile phase (pH 2.4). The concentration of each organic acid in the sample was calculated using a calibration curve obtained by using different concentrations for each acid. The peak corresponding to different acids was confirmed by comparison of the retention time with that of the standards [90].

### 3.12. Statistical Analysis

Germination experiments were performed in parallel using two biological replicates for each investigated legume at each sampling point in both treatment groups (with and without freezing pretreatment). For each biological replicate, all analytical measurements were conducted in technical triplicate (and in some cases, ≥3 technical measurements). Results are presented as mean values ± standard deviations of the biological replicates. Differences between treatment groups were analyzed using parametric tests (one-way ANOVA and Student’s *t*-test), with statistical significance set at *p* < 0.05. Pearson’s correlation coefficient (r) was used to assess the linear association between GABA content and GAD activity. Although the limited number of biological replicates prevents a robust formal assessment of normality assumptions, parametric tests were applied due to their widespread use and reasonable robustness in small biological datasets. All statistical analyses were performed using Microsoft Excel 2013 (Microsoft Corporation, Redmond, WA, USA).

## 4. Conclusions

This study provides a comprehensive evaluation of biochemical changes in commonly consumed legumes during a five-day germination period, with and without a freezing pretreatment. Germination consistently enhanced GABA levels, free polyphenols, free sugars, and organic acids across all legumes, while total protein content remained largely unaffected. Among the species examined, chickpeas exhibited the most pronounced response, showing substantial increases in GABA, selected phenolic compounds, antioxidant activity, and organic acids, thereby highlighting their potential as a promising matrix for the development of germinated functional foods. The principal strengths of this work include the comparative analysis of multiple legume species, the integrated assessment of nutritional and bioactive components, and the exploration of freezing pretreatment as a simple stress-based strategy to stimulate GABA accumulation. Nevertheless, the limited number of biological replicates and the use of a single genotype per species restrict broader generalization of the results. Accordingly, further studies incorporating expanded replication, improved statistical design, and multiple genotypes are required to validate and extend these findings.

## Figures and Tables

**Table 1:** Changes in the content of the major phenolic constituents (µg/g DW) in legumes after soaking and germination without freezing pretreatment.

Sample		Protocatechuic Acid	Vanillic Acid	Caffeic Acid	*p*-Coumaric Acid	Ferulic Acid	Salicylic Acid	(+)-Catechin	Rutin	Quercetin
US	mung beans	n.d.	n.d.	60.4 ^a^ ± 5.8	n.d.	94 ^a^ ± 8	n.d.	800 ^a^ ± 76	2500 ^a^ ± 200	92.0 ^a^ ± 8.2
PS		n.d.	n.d.	55.0 ^a^ ± 8.7	n.d.	110 ^b^ ± 11	n.d.	830 ^a^ ± 79	3200 ^b^ ± 280	120 ^b^ ± 10
120 h		n.d.	n.d.	17.2 ^b^ ± 2.1	n.d.	93 ^b^ ± 8.9	n.d.	1300 ^b^ ± 130	2800 ^ab^ ± 230	110 ^ab^ ± 12
US	Dobrudzha beans	n.d.	n.d.	n.d.	n.d.	n.d.	130 ^a^ ± 11	320 ^a^ ± 28	n.d.	79.6 ^a^ ± 6.4
PS		n.d.	n.d.	n.d.	n.d.	n.d.	130 ^a^ ± 11	320 ^a^ ± 30	n.d.	72 ^a^ ± 5.8
120 h		n.d.	n.d.	n.d.	n.d.	n.d.	120 ^a^ ± 12	360 ^a^ ± 35	n.d.	49 ^b^ ± 4.1
US	white beans	n.d.	n.d.	n.d.	n.d.	n.d.	n.d.	126 ^a^ ± 12	n.d.	51.2 ^a^ ± 4.5
PS		n.d.	n.d.	n.d.	n.d.	n.d.	n.d.	136 ^a^ ± 13	n.d.	73 ^b^ ± 7
120 h		n.d.	n.d.	n.d.	n.d.	n.d.	n.d.	181 ^b^ ± 15	n.d.	94 ^c^ ± 7
US	brown lentils	n.d.	n.d.	n.d.	190 ^a^ ± 16	103 ^a^ ± 10	108 ^a^ ± 11	450 ^a^ ± 50	59 ^a^ ± 4	n.d.
PS		n.d.	n.d.	n.d.	210 ^a^ ± 19	94 ^a^ ± 9	113 ^a^ ± 10	450 ^a^ ± 60	69 ^a^ ± 5	n.d.
120 h		n.d.	n.d.	n.d.	200 ^a^ ± 20	84 ^a^ ± 8	108 ^a^ ± 13	770 ^b^ ± 70	140 ^b^ ± 11	n.d.
US	red lentils	n.d.	130 ^a^ ± 12	n.d.	121 ^a^ ± 9	n.d.	290 ^a^ ± 26	390 ^a^ ± 40	n.d.	n.d.
PS		n.d.	110 ^a^ ± 15	n.d.	71.6 ^b^ ± 6.5	n.d.	99 ^b^ ± 7	350 ^a^ ± 34	n.d.	n.d.
120 h		n.d.	n.d.	n.d.	n.d.	n.d.	n.d.	393 ^a^ ± 32	n.d.	n.d.
US	chickpeas	23 ^a^ ± 2	n.d.	n.d.	n.d.	n.d.	n.d.	1200 ^a^ ± 120	n.d.	80 ^b^ ± 8
PS		24 ^a^ ± 8	n.d.	n.d.	n.d.	n.d.	n.d.	1380 ^ab^ ± 130	n.d.	44 ^a^ ± 8
120 h		64 ^b^ ± 6	n.d.	n.d.	n.d.	n.d.	n.d.	1530 ^b^ ± 150	n.d.	1540 ^c^ ± 150

Results are presented as mean values ± standard deviations; n.d.—not detectable. Statistical differences between time points or treatments were evaluated using one-way ANOVA, followed by Student’s *t*-test for pairwise comparisons. There are no significant differences among values marked with the same superscript letters in individual groups.

**Table 2:** Changes in the free sugar content (mg/100 g DW) of legumes after soaking and germination without freezing pretreatment (A) and with freezing pretreatment (B).

Sample		Fructose		Sucrose		Galactose		Maltose	
		mg/100 g DW		mg/100 g DW		mg/100 g DW		mg/100 g DW	
		A	B	A	B	A	B	A	B
US		49 ^b^ ± 4.5		n.d.		166 ^b^ ± 12		433 ^a^ ± 29	
PS	mung beans	36.4 ^a^ ± 3.6	21.3 ^a^ ± 2.2	n.d.	n.d.	166 ^b^ ± 11	106 ^a^ ± 9	540 ^b^ ± 47	297 ^a^ ± 51
24 h		62.9 ^c^ ± 5.6	26.8 ^a^ ± 2.3	n.d.	n.d.	185 ^b^ ± 15	187 ^b^ ± 22	650 ^b^ ± 58	749 ^c^ ± 51
48 h		215 ^db^ ± 20	210 ^d^ ± 20	n.d.	n.d.	186 ^b^ ± 17	233 ^c^ ± 21	720 ^c^ ± 69	893 ^d^ ± 63
72 h		403 ^ef^ ± 40	345 ^e^ ± 32	n.d.	n.d.	193 ^b^ ± 19	256 ^cd^ ± 24	880 ^d^ ± 78	1140 ^e^ ± 120
96 h		446 ^f^ ± 42	520 ^f^ ± 49	n.d.	n.d.	214 ^bc^ ± 21	271 ^d^ ± 26	970 ^de^ ± 90	1630 ^f^ ± 130
120 h		810 ^g^ ± 78	680 ^g^ ± 66	n.d.	n.d.	214 ^bc^ ±20	283 ^d^ ± 28	940 ± 87	1400 ^f^ ± 110
US		n.d.		n.d.		n.d.		850 ^b^ ± 80	
PS	Dobrudzha beans	n.d.	n.d.	n.d.	n.d.	n.d.	n.d.	610 ^a^ ± 60	500 ^a^ ± 46
24 h		n.d.	n.d.	n.d.	n.d.	n.d.	n.d.	1510 ^d^ ± 150	550 ^a^ ± 48
48 h		n.d.	n.d.	n.d.	n.d.	n.d.	n.d.	1940 ^e^ ± 180	860 ^b^ ± 79
72 h		n.d.	n.d.	n.d.	n.d.	n.d.	n.d.	2670 ^g^ ± 210	1200 ^c^ ± 90
96 h		n.d.	n.d.	n.d.	n.d.	n.d.	n.d.	2900 ^gh^ ± 310	2220 ^f^ ± 150
120 h		n.d.	n.d.	n.d.	n.d.	n.d.	n.d.	3080 ^h^ ± 290	2610 ^g^ ± 220
US		n.d.		n.d.		n.d.		830 ^a^ ± 76	
PS	white beans	n.d.	n.d.	n.d.	n.d.	n.d.	n.d.	1750 ^d^ ± 170	1110 ^b^ ± 110
24 h		n.d.	n.d.	n.d.	n.d.	n.d.	n.d.	2020 ^de^ ± 190	1270 ^bc^ ±120
48 h		n.d.	n.d.	n.d.	n.d.	n.d.	n.d.	2090 ^e^ ± 190	1310 ^c^ ± 130
72 h		n.d.	n.d.	n.d.	n.d.	n.d.	n.d.	2190 ^e^ ± 200	1460 ^c^ ± 140
96 h		n.d.	n.d.	n.d.	n.d.	n.d.	n.d.	2730 ^f^ ± 170	1470 ^c^ ± 140
120 h		n.d.	n.d.	n.d.	n.d.	n.d.	n.d.	3600 ^g^ ± 300	1730 ^d^ ± 110
US		49 ^a^ ±5		n.d.		n.d.		297 ^a^ ± 28	
PS	brown lentils	68.5 ^b^ ± 6.8	48 ^a^ ± 6	n.d.	n.d.	n.d.	n.d.	540 ^b^ ± 49	350 ^a^ ± 40
24 h		112 ^c^ ± 10	72 ^b^ ± 12	n.d.	n.d.	n.d.	n.d.	980 ^c^ ± 89	600 ^b^ ± 60
48 h		710 ^d^ ± 70	660 ^d^ ± 60	n.d.	n.d.	n.d.	n.d.	1080 ^c^ ± 100	620 ^b^ ± 60
72 h		2480 ^h^ ± 230	1860 ^g^ ± 170	n.d.	n.d.	n.d.	n.d.	1180 ^cd^ ± 110	1350 ^d^ ± 130
96 h		1550 ^i^ ± 140	1010 ^f^ ±100	n.d.	n.d.	n.d.	n.d.	3560 ^f^ ± 290	1840 ^e^ ± 180
120 h		790 ^e^ ± 76	607 ^d^ ± 60	n.d.	n.d.	n.d.	n.d.	3260 ^f^ ± 280	1100 ^c^ ± 110
US		44 ^a^ ± 3		n.d.		n.d.		480 ^e^ ± 40	
PS	red lentils	120 ^b^ ± 14	105 ^b^ ± 11	n.d.	n.d.	n.d.	n.d.	220 ^c^ ± 20	60.9 ^a^ ± 11.7
24 h		170 ^c^ ± 16	118 ^b^ ± 11	n.d.	n.d.	n.d.	n.d.	240 ^c^ ± 22	80.9 ^a^ ± 10.8
48 h		170 ^c^ ± 16	220 ^d^ ± 19	n.d.	n.d.	n.d.	n.d.	240 ^c^ ± 23	150 ^b^ ± 13
72 h		190 ^cd^ ± 17	310 ^e^ ± 47	n.d.	n.d.	n.d.	n.d.	240 ^c^ ± 24	420 ^d^ ± 41
96 h		180 ^c^ ± 17	280 ^e^ ± 25	n.d.	n.d.	n.d.	n.d.	1140 ^g^ ± 110	1150 ^g^ ± 105
120 h		180 ^c^ ± 16	243 ^d^ ± 21	n.d.	n.d.	n.d.	n.d.	790 ^f^ ± 100	940 ^ffg^ ± 110
US		130 ^b^ ± 12		160 ^c^ ± 14		n.d.		1050 ^c^ ± 100	
PS	chickpeas	49 ^a^ ± 10	97 ^b^ ± 20	130 ^b^ ± 12	150 ^bc^ ± 20	n.d.	n.d.	400 ^a^ ± 34	709 ^b^ ± 71
24 h		300 ^c^ ± 26	458 ^d^ ± 44	230 ^d^ ± 21	350 ^e^ ± 28	n.d.	n.d.	1390 ^d^ ± 130	1940 ^f^ ± 190
48 h		770 ^ef^ ± 71	670 ^e^ ± 62	210 ^d^ ± 20	560 ^e^ ± 46	n.d.	n.d.	1645 ^e^ ± 160	2320 ^f^ ± 200
72 h		950 ^g^ ± 89	690 ^e^ ± 64	120 ^b^ ± 11	170 ^c^ ± 30	n.d.	n.d.	1750 ^e^ ± 170	3390 ^g^ ± 310
96 h		900 ^fg^ ± 67	740 ^ef^ ± 66	69 ^a^ ± 6	140 ^b^ ± 12	n.d.	n.d.	1870 ^ef^ ± 160	2360 ^f^ ± 210
120 h		810 ^f^ ± 77	670 ^e^ ±58	55 ^a^ ± 9	71 ^a^ ± 10	n.d.	n.d.	1450 ^d^ ± 130	2040 ^ef^ ± 200

Results are presented as mean values ± standard deviations; n.d.—not detectable. Statistical differences between time points or treatments were evaluated using one-way ANOVA, followed by Student’s *t*-test for pairwise comparisons. There are no significant differences among values marked with the same superscript letters in individual groups.

**Table 3:** Changes in organic acids content (mg/100 g DW) in legumes during germination without freezing pretreatment (A) and with freezing pretreatment (B).

Samples		Oxalic Acid		Tartaric Acid		Quinic Acid		Shikimic Acid		Fumaric Acid		Malic Acid	
		mg/100 g DW		mg/100 g DW		mg/100 g DW		mg/100 g DW		mg/100 g DW		mg/100 g DW	
		A	B	A	B	A	B	A	B	A	B	A	B
US		33 ^a^ ± 8		168 ^a^ ± 15		4950 ^a^ ± 420		n.d.		4.4 ^ab^ ± 0.4		178 ^a^ ± 16	
PS	mung beans	77 ^b^ ± 7	46 ^a^ ± 14	224 ^b^ ± 21	168 ^a^ ±15	4760 ^a^ ± 420	4910 ^a^ ± 430	n.d.	n.d.	3.5 ^ab^ ± 0.9	3.5 ^ab^ ± 0.6	186 ^a^ ± 17	202 ^ab^ ± 21
24 h		87 ^b^ ±8	79 ^b^ ± 7	305 ^c^ ± 28	234 ^b^ ± 21	5330 ^b^ ± 520	5330 ^b^ ± 500	n.d.	n.d.	2.7 ^a^ ± 0.1	5.7 ^b^c ± 0.5	232 ^b^ ± 21	211 ^ab^ ± 21
48 h		177 ^c^ ± 16	158 ^c^ ± 15	315 ^c^ ± 30	285 ^c^ ± 26	5620 ^bc^ ± 530	6180 ^c^ ± 590	n.d.	n.d.	4.9 ^b^ ± 0.7	5.4 ^b^ ± 0.7	243 ^b^ ± 22	215 ^ab^ ± 21
72 h		279 ^de^ ± 26	215 ^cd^ ± 20	482 ^d^ ± 40	386 ^d^ ± 31	5770 ^bc^ ± 550	6760 ^c^ ± 630	n.d.	n.d.	4.7 ^b^ ± 0.8	5.8 ^bc^ ± 0.9	252 ^bc^ ± 23	234 ^b^ ± 23
96 h		266 ^de^ ± 21	281 ^de^ ± 26	476 ^de^ ± 36	473 ^de^ ± 45	5710 ^bc^ ± 530	7510 ^d^ ± 690	n.d.	n.d.	5.1 ^b^ ± 1.1	6.3 ^bc^ ± 0.9	256 ^bc^ ± 23	243 ^b^ ± 24
120 h		304 ^e^ ± 31	425 ^f^ ± 38	573 ^e^ ± 52	804 ^f^ ± 80	7680 ^d^ ± 620	8730 ^e^ ± 710	n.d.	n.d.	7.6 ^b^ ± 0.2	9.7 ^d^ ± 1.1	295 ^c^ ±26	339 ^d^ ± 32
US		77 ^a^ ± 8		194 ^a^ ± 19		5100 ^cd^ ± 600		5.1 ^a^ ± 0.5		n.d.		n.d.	
PS	Dobrudzha beans	120 ^b^ ± 11	124 ^b^ ± 12	295 ^b^ ± 28	190 ^a^ ± 18	2410 ^a^ ± 210	2320 ^a^ ± 230	8.8 ^b^ ± 0.8	5.6 ^a^ ± 0.9	n.d.	n.d.	n.d.	n.d.
24 h		133 ^b^ ± 12	126 ^b^ ± 12	305 ^b^ ± 30	258 ^b^ ± 28	4100 ^c^ ± 390	2310 ^a^ ± 220	8.6 ^b^ ± 1.2	9.8 ^b^ ± 1.2	n.d.	n.d.	n.d.	n.d.
48 h		131 ^b^ ± 12	139 ^b^ ± 13	324 ^bc^ ± 31	284 ^b^ ± 27	5210 ^cd^ ± 500	3490 ^b^ ± 320	8.1 ^b^ ± 1.2	8.7 ^b^ ± 0.9	n.d.	n.d.	n.d.	n.d.
72 h		133 ^b^ ± 13	199 ^c^ ± 18	316 ^bc^ ± 32	370 ^bc^ ± 31	5710 ^d^ ± 520	4900 ^c^ ± 420	8.7 ^b^ ± 0.7	10.1 ^c^ ± 0.9	n.d.	n.d.	n.d.	n.d.
96 h		165 ^c^ ± 15	254 ^d^ ± 24	419 ^c^ ± 39	458 ^c^ ± 39	6120 ^de^ ± 610	6650 ^e^ ± 640	9.5 ^bc^ ± 0.9	10.2 ^c^ ± 0.6	n.d.	n.d.	n.d.	n.d.
120 h		191 ^c^ ± 18	552 ^e^ ± 51	349 ^bc^ ± 32	364 ^bc^ ± 33	6180 ^de^ ± 610	7310 ^f^ ± 700	10 ^c^ ± 1.1	12.5 ^d^ ± 2.1	n.d.	n.d.	n.d.	n.d.
US		65 ^ab^ ±6		214 ^ab^ ± 20		4300 ^d^ ± 400		3.8 ^a^ ± 0.1		2.1 ^a^ ± 0.1		n.d.	
PS	white beans	60 ^a^ ±6	56 ^a^ ± 8	230 ^b^ ± 20	185 ^a^ ± 17	4350 ^d^ ± 400	3150 ^a^ ± 300	4.5 ^ab^ ± 0.8	3.6 ^a^ ± 0.5	2.0 ^a^ ± 0.5	2.9 ^ab^ ±0.6	n.d.	n.d.
24 h		90 ^c^ ± 8	72 ^b^ ± 6	261 ^c^ ± 20	187 ^a^ ± 17	4340 ^d^ ± 400	3130 ^a^ ± 300	6.7 ^b^ ± 0.7	4.4 ^ab^ ± 0.8	3.7 ^b^ ± 0.8	3.4 ^b^ ± 0.6	n.d.	n.d.
48 h		125 ^d^ ± 10	116 ^d^ ± 10	254 ^a^c ± 18	206 ^ab^ ± 20	4460 ^d^ ± 400	3310 ^b^ ± 300	9.3 ^c^ ± 1.2	5.7 ^b^ ± 1.1	3.5 ^b^ ± 0.5	3.3 ^b^ ± 0.6	n.d.	n.d.
72 h		174 ^e^ ± 15	116 ^d^ ± 10	372 ^d^ ± 27	222 ^b^ ± 21	5350 ^e^ ± 500	3500 ^b^ ± 300	11.3 ^cd^ ± 0.5	6.0 ^b^ ± 0.7	5.3 ^c^ ± 0.5	3.4 ^b^ ± 0.6	n.d.	n.d.
96 h		171 ^e^ ± 15	117 ^d^ ± 10	449 ^e^ ± 35	225 ^b^ ± 24	7830 ^g^ ± 700	3400 ^b^ ± 300	12.6 ^d^ ± 0.8	6.4 ^b^ ± 0.8	9.2 ^d^ ± 0.8	3.1 ^b^ ± 0.5	n.d.	n.d.
120 h		406 ^g^ ± 31	263 ^f^ ± 25	967 ^af^ ± 84	453 ^e^ ± 36	7090 ^f^ ± 700	3740 ^c^ ± 300	25.1 ^e^ ± 1.7	12.5 ^d^ ± 2.1	15.2 ^e^ ± 0.9	5.6 ^c^ ± 0.8	n.d.	n.d.
US		128 ^a^ ± 13		241 ^a^ ± 22		2600 ^a^ ± 260		n.d.		n.d.		n.d.	
PS	brown lentils	252 ^c^ ± 25	181 ^b^ ± 16	547 ^c^ ± 50	459 ^b^ ± 39	4420 ^b^ ± 410	3230 ^b^ ± 310	n.d.	n.d.	n.d.	n.d.	n.d.	n.d.
24 h		285 ^c^ ±26	205 ^b^ ± 19	555 ^c^ ± 51	507 ^bc^ ± 41	4485 ^b^ ± 410	3310 ^b^ ± 220	n.d.	n.d.	n.d.	n.d.	n.d.	n.d.
48 h		461 ^e^ ± 42	376 ^d^ ± 32	648 ^d^ ± 62	596 ^cd^ ± 49	4590 ^c^ ± 430	3370 ^b^ ± 220	n.d.	n.d.	n.d.	n.d.	n.d.	n.d.
72 h		841 ^g^ ± 83	472 ^e^ ± 41	798 ^e^70	766 ^e^ ± 71	4650 ^c^ ± 460	3510 ^c^ ± 230	n.d.	n.d.	n.d.	n.d.	n.d.	n.d.
96 h		896 ^g^ ± 82	561 ^f^ ± 52	959 ^f^ ± 89	662 ^d^ ± 63	4570 ^c^ ± 420	2950 ^a^ ± 280	n.d.	n.d.	n.d.	n.d.	n.d.	n.d.
120 h		834 ^g^ ± 82	623 ^f^ ± 60	984 ^f^ ± 96	530 ^c^ ± 51	4560 ^c^ ± 440	2470 ^a^ ± 220	n.d.	n.d.	n.d.	n.d.	n.d.	n.d.
US		173 ^a^ ± 17		316 ^c^ ± 30		1210 ^d^ ± 100		n.d.		n.d.		n.d.	
PS	red lentils	170 ^a^ ± 17	147 ^a^ ± 12	118 ^a^ ± 10	132 ^a^ ± 12	773 ^a^ ± 70	947 ^b^ ± 90	n.d.	n.d.	n.d.	n.d.	n.d.	n.d.
24 h		207 ^b^ ± 20	244 ^c^ ± 21	196 ^b^ ± 18	226 ^b^ ± 21	824 ^ab^ ± 80	1080 ^c^ ± 110	n.d.	n.d.	n.d.	n.d.	n.d.	n.d.
48 h		206 ^b^ ± 19	226 ^bc^ ± 24	410 ^d^ ± 40	311 ^c^ ± 30	1716 ^e^ ± 168	1320 ^d^ ± 120	n.d.	n.d.	n.d.	n.d.	n.d.	n.d.
72 h		226 ^bc^ ± 20	344 ^d^ ± 32	463 ^d^ ± 45	430 ^d^ ± 40	2300 ^f^ ± 210	1450 ^d^ ± 140	n.d.	n.d.	n.d.	n.d.	n.d.	n.d.
96 h		611 ^f^ ± 57	365 ^d^ ± 32	426 ^d^ ± 40	435 ^d^ ± 40	2930 ^g^ ± 260	3830 ^e^ ± 310	n.d.	n.d.	n.d.	n.d.	n.d.	n.d.
120 h		701 ^g^ ± 62	546 ^e^ ± 51	570 ^e^ ± 50	534 ^e^ ± 50	2670 ^fg^ ± 250	3060 ^g^ ± 270	n.d.	n.d.	n.d.	n.d.	n.d.	n.d.
US		37 ^b^ ± 3		n.d.		4260 ^c^ ± 310		n.d.		8.4 ^de^ ± 0.2		n.d.	
PS	chickpeas	26 ^a^ ± 3	33 ^ab^ ± 3	n.d.	n.d.	2840 ^a^ ± 280	4040 ^c^ ± 370	n.d.	n.d.	2.9 ^a^ ± 0.6	4.2 ^b^ ± 0.8	n.d.	n.d.
24 h		65 ^c^ ± 6	98 ^d^ ± 9	n.d.	n.d.	3460 ^b^ ± 310	4500 ^cd^ ± 400	n.d.	n.d.	5.7 ^c^ ± 0.8	6.9 ^ce^ ± 0.7	n.d.	n.d.
48 h		71 ^c^ ± 7	114 ^e^ ± 11	n.d.	n.d.	3180 ^ab^ ± 320	4500 ^cd^ ± 400	n.d.	n.d.	5.9 ^c^ ± 0.6	7.2 ^cd^ ± 1.1	n.d.	n.d.
72 h		75 ^c^ ± 7	110 ^e^ ± 10	n.d.	n.d.	4010 ^c^ ± 350	4900 ^de^ ± 460	n.d.	n.d.	5.6 ^c^ ± 0.5	9.1 ^e^ ± 0.8	n.d.	n.d.
96 h		116 ^e^ ± 10	182 ^f^ ± 18	n.d.	n.d.	4060 ^c^ ± 320	5780 ^e^ ± 570	n.d.	n.d.	7.5 ^d^ ± 0.8	12.6 ^f^ ± 1.2	n.d.	n.d.
120 h		164 ^f^ ± 15	188 ^f^ ± 18	n.d.	n.d.	6890 ^f^ ± 580	6370 ^ef^ ± 600	n.d.	n.d.	13.0 ^f^ ± 1.8	16.1 ^g^ ± 1.2	n.d.	n.d.

Results are presented as mean values ± standard deviations; n.d.—not detectable. Statistical differences between time points or treatments were evaluated using one-way ANOVA, followed by Student’s *t*-test for pairwise comparisons. There are no significant differences among values marked with the same superscript letters in individual groups.

## Data Availability

Data is contained within the article or Supplementary Materials.

## Supplementary Materials

The following supporting information can be downloaded at: https://www.mdpi.com/article/10.3390/plants15020242/s1, Figure S1: Relation between biuret absorbance value and Kjeldahl protein for all legume samples; Table S1. Changes in GABA, total protein, total polyphenols and ORAC in legumes during germination.

## Abbreviations

The following abbreviations are used in this manuscript:

GABA	Gamma-aminobutyric acid
ORAC	Oxygen Radical Absorbance Capacity
GAD	Glutamate decarboxylase
HPLC	High-performance Liquid Chromatography
FTPC	Free total polyphenol content
