# Supplementary material for: Germination Triggers Substantial Changes in GABA, Polyphenol, Sugar, and Organic Acid Content of Commonly Consumed Legumes
Source: Plants (Basel). 2026 Jan 13;15(2):242. doi: 10.3390/plants15020242 (PMC12844749; doi:10.3390/plants15020242)
Supplement: Supplementary file 1 [file plants-15-00242-s001.zip › plants-3934942-supplementary.pdf]

Germination triggers substantial changes in GABA, polyphenol, sugar, and organic acid content of commonly consumed legumes

Daniela Pencheva [1], Desislava Teneva [1,2], Zornica Todorova [1], Manol Ognyanov [1,2], Ani Petrova [1], Vasil Georgiev [3], Mariya Pimpilova [1] and Petko Denev [1,2,*]

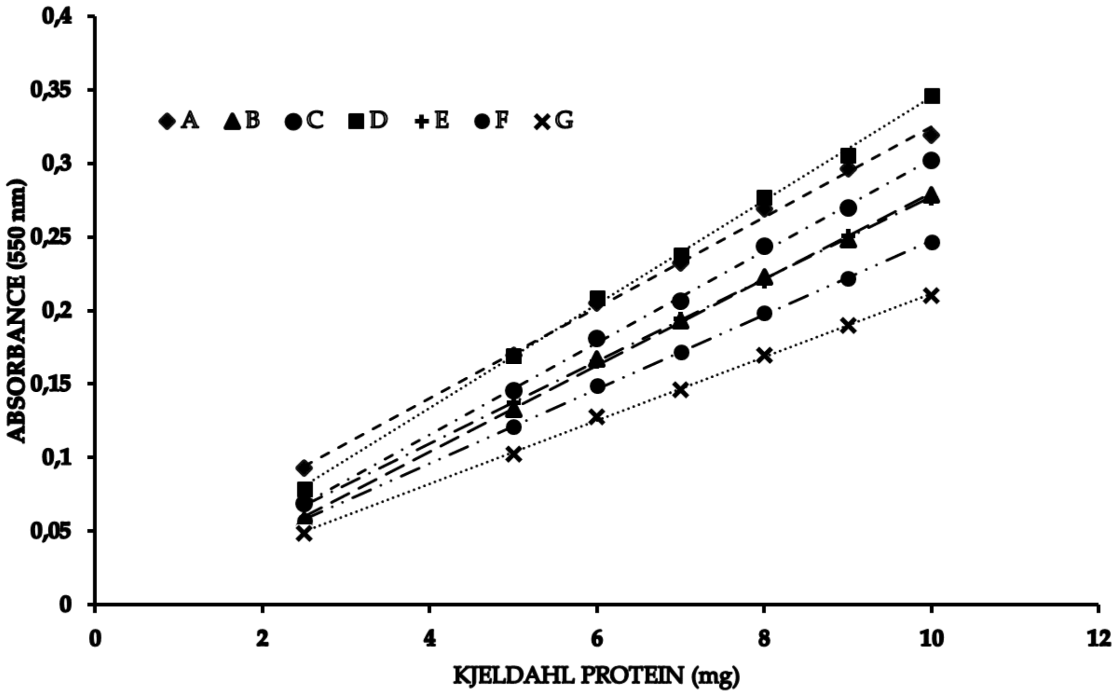

Figure S1. Relation between biuret absorbance value and Kjeldahl protein for all legume samples: A: mung beans (Y=0.0308.X+0.0176, R^2=0.9976); B: Dobrudzha beans (Y=0.0293.X-0.0129, R^2=0.9988); C: white beans (Y=0.0311.X-0.0088, R^2=0.9992); D: Smilyan beans (Y=0.0352.X-0.0072, R^2=0.999); E: brown lentils (Y=0.0279.X-0.0019, R^2=0.9998); F: red lentils (Y=0.0253.X-0.0049, R^2=0.9995); G: chickpeas (Y=0.0216.X-0.0044, R^2=0.9993).

Table S1. Changes in GABA, total protein, total polyphenols and ORAC in legumes after soaking and germination

Samples		GABA mg/100g DW		Total protein g/100g DW		Total polyphenols mg GAE/100g DW		ORAC µmol TE/g DW	
		A	B	A	B	A	B	A	B
US	Mung beans	7±0.8		21±1.2		366±25.1		144±16.5	
PS		14±1.9	8±1.8	22±1.9	20±1.1	366±29.8	314±30.0	139±13.7	105±10.1
24h		15±2.1	26±2.3	23±2.3	23±1.5	339±36.5	302±31.2	121±12.5	96±8.5
48h		25±2.6	27±2.8	22±2.5	21±1.9	290±31.2	256±36.0	134±16.2	100±11.2
72h		28±2.5	27±2.4	20±2.4	19±2.0	223±28.5	246±35.2	119±10.4	98±9.4
96h		19±2.7	23±2.8	18±3.0	19±2.0	272±30.5	262±38.1	122±10.5	114±11.2
120h		17±2.8	23±2.6	18±3.1	19±1.9	308±34.6	307±36.5	127±12.3	114±10.7
US	Dobrudzha beans	15±0.6		19±1.0		70±5.6		47±4.1	
PS		24±1.2	11±1.4	18±2.0	19±1.3	88±6.4	60±6.9	47±4.5	48±4.8
24h		21±1.0	12±1.3	19±2.1	20±1.5	83±5.5	74±6.1	51±5.0	49±5.4
48h		22±1.5	12±1.8	19±2.5	20±1.9	75±4.9	82±6.9	49±3.8	53±4.3
72h		23±1.4	13±1.9	20±2.9	19±1.4	90±6.1	83±7.0	50±4.1	53±6.5
96h		13±1.0	16±1.8	17±3.3	19±3.1	91±7.2	84±7.3	55±5.5	56±5.1
120h		19±1.2	12±2.0	22±2.8	21±3.2	93±7.3	105±7.0	56±4.2	59±6.2
US	White beans	23±0.9		20±0.8		48±4.5		68±5.8	
PS		29±1.1	36±1.2	21±1.6	21±1.2	56±6.8	65±7.2	47±4.2	65±5.8
24h		33±1.4	60±1.0	21±1.5	23±1.5	67±5.4	81±6.5	77±8.1	72±7.0
48h		37±3.1	61±4.8	22±2.0	23±1.1	85±8.5	96±9.0	83±8.2	77±6.9
72h		46±3.7	32±4.9	21±2.2	23±1.7	107±11.3	106±12.3	99±8.7	71±6.8
96h		43±3.9	37±5.0	21±2.8	22±1.5	152±14.2	105±14.5	111±13.8	87±7.6
120h		42±3.7	45±5.6	21±2.5	22±2.0	160±13.5	188±16.2	124±15.2	101±11.3
US	Brown lentils	24±0.9		27±2.0		130±6.5		67±5.5	
PS		24±1.8	41±2.2	29±2.1	29±1.5	138±12.5	133±13.8	69±6.0	61±6.4
24h		21±2.1	31±3.0	27±2.5	29±2.2	146±11.6	137±16.5	68±6.4	62±5.8
48h		36±2.8	57±3.9	28±2.2	28±1.8	149±15.8	159±18.0	76±8.1	61±6.0
72h		40±3.8	59±4.9	27±3.3	28±2.5	171±17.1	164±19.5	100±10.4	63±7.1
96h		42±5.0	59±5.6	28±3.4	29±2.6	174±17.0	173±20.3	122±14.1	66±6.6
120h		62±6.9	63±7.2	28±3.5	29±3.4	171±16.7	183±24.6	128±14.6	63±6.4
US	Red lentils	23±0.8		28±1.2		153±8.2		54±4.7	
PS		24±2.1	14±1.6	29±1.5	30±1.3	153±10.5	158±10.3	42±4.5	42±3.6
24h		35±3.5	30±3.2	29±1.8	29±1.7	150±15.6	159±14.0	48±4.2	44±3.8
48h		15±1.5	19±1.8	30±2.3	30±2.2	185±18.7	156±13.2	51±6.1	44±4.0
72h		20±2.1	6±0.8	29±2.5	30±2.8	192±19.8	158±14.5	47±3.7	39±4.1
96h		47±4.8	6±0.7	28±3.0	28±3.1	382±28.0	166±15.6	129±12.7	37±3.2
120h		74±8.1	7±0.9	25±3.6	27±3.9	336±38.0	200±20.0	122±12.2	45±5.2
US	Chickpeas	12±1.0		23±1.2		83±4.6		45±5.7	
PS		13±1.8	13±1.9	25±3.4	25±2.9	138±13.1	139±13.1	49±3.7	47±4.5
24h		21±2.1	24±2.8	25±3.1	25±3.0	126±12.5	163±10.6	50±4.8	54±5.7
48h		40±4.5	68±6.4	25±3.8	24±2.8	167±14.5	169±13.8	49±4.7	60±5.8
72h		111±10.8	114±14.5	26±3.6	25±3.9	229±18.9	179±20.1	66±5.2	65±6.6
96h		174±18.9	159±16.4	26±3.8	22±3.8	253±23.4	214±28.9	78±8.1	78±8.2
120h		208±20.3	210±23.8	26±4.0	26±3.2	278±32.3	206±30.6	83±8.7	75±7.7

* Results are presented as mean values ± standard deviations; without freezing pretreatment (A) and with freezing pretreatment (B)
